# Occurrence and Molecular Characteristics of *Mcr-1*-Positive *Escherichia coli* from Healthy Meat Ducks in Shandong Province of China

**DOI:** 10.3390/ani10081299

**Published:** 2020-07-29

**Authors:** Fengzhi Liu, Ruihua Zhang, Yupeng Yang, Hanqing Li, Jingyu Wang, Jingjing Lan, Pengfei Li, Yanli Zhu, Zhijing Xie, Shijin Jiang

**Affiliations:** 1 College of Veterinary Medicine, Shandong Agricultural University, Taian 271000, China; lfz156@163.com (F.L.); ruirui041127@126.com (R.Z.); 18865485081@163.com (Y.Y.); lihqnqing13146@163.com (H.L.); jywang676@163.com (J.W.); jjlan1024@163.com (J.L.); p.li@erasmusmc.nl (P.L.); ylz@sdau.edu.cn (Y.Z.); xiezhj@sdau.edu.cn (Z.X.); 2 Shandong Provincial Key Laboratory of Animal Biotechnology and Disease Control and Prevention, Taian 271000, China

**Keywords:** fecal *Escherichia coli*, *mcr-1*, plasmid, healthy meat duck

## Abstract

**Simple Summary:**

Colistin has been used as a growth promotant in livestock feed for many years. To date, there are few reports about the prevalence and molecular characteristics of fecal *Escherichia coli* bearing *mcr-1* in the meat ducks. In this study, among 120 fecal *Escherichia coli* strains isolated from healthy meat ducks, a total of nine *mcr-1*-containing *E. coli* strains were identified and two were identified as extra-intestinal pathogenic *E. coli*. The 9 *mcr-1*-bearing *E. coli* isolates were clonally unrelated, carried two different genetic contexts of *mcr-1*, and the colistin-resistant phenotype of them was successfully transferred to the recipient strains. These results highlight that healthy meat duck is a potential reservoir for multidrug resistant *mcr-1*-containing *E. coli* strains.

**Abstract:**

Colistin has been used as a growth promotant in livestock feed for many years. In China, *mcr-1*-positive *Escherichia coli* strains have been isolated from humans, chickens, and pigs. To date, there are few reports about the prevalence and molecular characteristics of fecal *E. coli* bearing *mcr-1* in the meat ducks. In this study, the prevalence of *mcr-1* gene was investigated among 120 fecal *E. coli* strains isolated from healthy meat ducks in Shandong province of China between October 2017 and February 2018. A total of nine *mcr-1*-containing *E. coli* strains were identified and two were identified as extra-intestinal pathogenic *E. coli* (ExPEC) among them. The clonal relationship of the nine *E. coli* strains was determined by multilocus sequencing typing (MLST) and pulsed field gel electrophoresis (PFGE), and the results indicated that all *mcr-1*-carrying isolates were clonally unrelated. Two different genetic contexts of *mcr-1* were identified among these isolates. Colistin-resistant phenotype of all the isolates was successfully transferred to the recipient strains by conjugation experiments and seven transconjugants carried a single plasmid. The *mcr-1* was located on three replicon plasmids: IncI2 (n = 4), IncFII (n = 2) and IncN (n = 1). Complete sequence analysis of a representative plasmid pTA9 revealed that it was strikingly similar with plasmid pMCR1-IncI2 of *E. coli*, plasmid pHNSHP45 of *E. coli*, and plasmid pWF-5-19C of *Cronobacter sakazakii*, implying that pTA9-like plasmids may be epidemic plasmids that mediate the spread of *mcr-1* among *Enterobacteriaceae*. These results highlight that healthy meat duck is a potential reservoir for multidrug resistant *mcr-1*-containing *E. coli* strains.

## 1. Introduction

Avian pathogenic *Escherichia coli* (APEC), a subgroup of extra-intestinal pathogenic *E. coli* (ExPEC), can cause severe disease characterized by perihepatitis, pericarditis, and airsacculitis, which results in economic and welfare costs in the poultry industry worldwide [1]. There are similar virulence genes between APEC strains and the ExPEC strains in humans [2]. Via the food chain, the multidrug resistant (MDR) APEC strains can transfer from poultry to man, which not only increases the difficulty of treating animal diseases, but also poses a serious threat to human health [3]. 

As a polymyxin antibacterial agent, colistin is considered as the last-resort drug with excellent bactericidal activity against multidrug-resistant Gram-negative pathogens in humans [4]. However, the recent emergence of *mcr*-like genes (*mcr-1* to *mcr-10*) potentially threatens the clinical effectiveness of colistin [5,6,7]. These *mcr* genes have been disseminated to more than 40 countries across at least five continents in multiple ecosystems and traced to more than 11 bacterial species [8,9]. The worldwide distribution of *mcr-1* gene strongly indicates a potential food-chain-based spread route [10]. Many studies showed that the prevalent dissemination of the *mcr-1* gene relied on transfer by conjugative plasmids such as pHNSHP45, pECJS-B65–33, and pECJS-61–63 [8,9,11]. 

The intestinal flora of the food animals and humans is a reservoir for antibiotic resistance genes, and the resistant genes can spread from food animals to humans by commensal flora [12,13]. In China, *mcr-1*-positive *E. coli* strains have been isolated from humans, chickens, and pigs [14]. To date, prevalence and molecular characteristics of many viral and bacterial pathogens has been identified in Chinese duck flocks [15,16,17,18,19,20,21], but there are few reports about the prevalence and molecular characteristics of fecal *E. coli* bearing *mcr-1* from the meat ducks [22,23,24]. In this study, we isolated *E. coli* strains from the feces of healthy meat ducks in Shandong province of China, and investigated the occurrence and molecular characteristics of the *mcr-1*-positive *E. coli* strains.

## 2. Materials and Methods 

### 2.1. Bacterial Isolate

From October 2017 to February 2018, a total of 120 cloacal swabs were collected from healthy meat ducks from 12 duck farms in Shandong province, China. The cloacal swabs were immediately put into Luria-Bertani (LB) broth and incubated for 24 h at 37 °C. All samples were seeded on selective MacConkey agar plates. Bright pink, round, and smooth surface *E. coli* colonies were picked on selective plates for further analysis. The *E. coli* isolates were identified through 16S rDNA sequence analysis, and the 16S rDNA primers were designed in this study (Table 1). 

### 2.2. Antimicrobial Susceptibility Testing

The minimum inhibitory concentrations (MICs) of tetracycline, fosfomycin, colistin, gentamicin, imipenem, ciprofloxacin, cefotaxime, amikacin, and florfenicol for the *E. coli* isolates picked on the plates and transconjugants were tested by the broth dilution method and interpreted according to the Clinical and Laboratory Standards Institute [25,26]. The colistin breakpoint (≥2 μg/mL) was used according to the European Committee on Antimicrobial Susceptibility Testing guidelines [27]. *E. coli* ATCC 25,922 was used as the quality-control strain.

### 2.3. Molecular Detection

All colistin resistant *E. coli* strains and their transconjugants were screened for *mcr-1* gene by polymerase chain reaction (PCR) assays [14]. According to the surrounding structure of pTA9, the primers of *nikB* and *top* gene were designed to determine the genetic environment of the *mcr-1* gene (Table 1). The resistance genes (*floR*, *tet*(A), β-Lactamase, *rmtB*, and *fosA3*) and virulence-associated genes were analyzed for the *mcr-1*-containing *E. coli* strains and their transconjugants by PCR (Table S1) [28,29,30,31]. The strains were classified as ExPEC if they carried at least two of five key virulence genes: *papA* and/or *papC* (pyelonephritis-associated pili A/C, counted as 1: P fimbriae), *sfa*/*foc* (S/F1C fimbriae), *afa*/*dra* (Afimbrial/Dr-binding adhesins), *iutA* (aerobactin system), and kps*M* II (group 2 capsules) [32].

### 2.4. Molecular Typing

XbaI-PFGE was performed as described previously [33] using the CHEF-MAPPER System (Bio-Rad Laboratories, Hercules, CA, USA). Phylogenetic analysis of PFGE patterns was performed using the PyElph software version 1.4 [34]. The UPGMA method was used for clustering. *Mcr*-1-positive strains were studied by multilocus sequence typing (MLST) as previously described [35]. Phylogenetic classification was performed using a triplex PCR reaction [36].

### 2.5. Conjugation Assays

Conjugation experiments were performed using azide resistant *E. coli* J53 as the recipient [37]. Transconjugants were selected on agar containing 200 mg/L azide and 2 mg/L colistin and confirmed by enterobacterial repetitive intergenic consensus (ERIC)-PCR method [38]. 

### 2.6. Plasmid Characterization

*Mcr-1*-containing plasmids were sized by the S1 nuclease pulsed field gel electrophoresis (S1-PFGE) [33]. A single plasmid carried by transconjugants was used for plasmid analysis. The replicon types of plasmids were determined by PCR-based replicon typing (PBRT) [39]. A representative *mcr-1*-harboring plasmid, pTA9, was extracted using the Qiagen Large Construct kit (Qiagen, Hilden, Germany) and sequenced using the Illumina MisSeq system using prepared paired-end 2 × 300 bp libraries. The coverage of the plasmid is 200×. Raw data was assembled using the SPAdes Genome Assembler (http://cab.spbu.ru/software/spades/) and SSPACE (version 3.0). Gap was closed with PCR and Sanger sequencing. The plasmid was annotated using the RAST tool (http://rast.nmpdr.org/). 

### 2.7. Ethics Statement

All animal experiments were carried out in accordance with guidelines issued by the Shandong Agricultural University Animal Care and Use Committee (approval number, SDAUA-2017-043). 

## 3. Results and Discussion

### 3.1. Identification of Mcr-1-Carrying E. coli Isolates 

In this study, a total of 120 fecal *E. coli* strains were isolated from healthy meat ducks from October 2017 to February 2018. Among them, only nine isolates (7.5%, 9/120) were resistant to colistin and identified as positive for *mcr-1* gene by PCR amplification and sequencing. In China, high *mcr-1* gene carriage rates (about 15% to 30%) were observed in *E. coli* isolates collected from poultry and pigs between 2011 to 2016 [14,40,41]. Colistin had been commonly used as a growth promotant in livestock feed for many years and had been banned from April 2017 in China. However, the samples in the above-mentioned studies were collected before the ban was issued [14,40,41]. The samples in this study were collected after the ban was issued. So, we speculated that the ban of colistin in animal feed might be the main reason why the low frequency of *mcr-1* gene was found in fecal *E. coli* isolates in this study. 

### 3.2. Antimicrobial Resistance Patterns and Resistance Genes

In this study, all of the 9 *mcr*-1-bearing *E. coli* isolates were MDR strains (resistance to antibiotics of at least three classes). Among them, 9, 8, 8, and 7 isolates were resistant to tetracycline, cefotaxime, ciprofloxacin, and florfenicol respectively, but all were susceptible to imipenem (Table 2). *Mcr-1* is usually found to coexist with other resistance genes (extended-spectrum β-lactam, *floR*, and *tet*(A)) in bacteria [42,43,44]. In this study, 6, 5, 5, and 2 of the nine *mcr-1*-bearing *E. coli* isolates harbored *floR*, *bla*_CTX-M_, *bla*_TEM-1,_ and *tet*(A) genes, respectively (Table 2). The association with other resistance genes is likely to favor the dissemination of *mcr-1* by co-selection, since cephalosporins, florfenicol, and tetracycline are used extensively in animal husbandry in China. 

### 3.3. Phylogenetic Groups and Virulence Genes

All of the nine *mcr-1*-bearing *E. coli* isolates contained virulence genes, and the *iutA* (aerobactin acquisition) gene was identified in 6 ones (Table 2). Two of the nine *E. coli* isolates, namely TA9 and TA103 carrying both *iutA* and *papC* genes were identified as ExPEC according to the standard [32] (Table 2). The presence of *mcr-1*-harboring ExPEC isolates in healthy meat ducks posed a serious health threat to consumers. Fortunately, no virulence gene was co-transferred with *mcr-1* gene to the recipient (Table 3). To the best of our knowledge, this is the first report about *mcr-1*-positive ExPEC isolates identified from healthy meat animals.

Phylogenetic group analysis revealed that seven (77.8%) of the nine *mcr-1*-bearing *E. coli* isolates belonged to group A and the other two isolates were classed into group D and B1, respectively (Table 2). Similar results were found in the fecal *E. coli* isolates from chickens in Australia, which were classed into group A, D, B1, and B2, and group A was dominant [45]. The two ExPEC isolates (TA9 and TA103) respectively belonged to groups A and D, which was similar to the result that ExPEC isolates from retail chicken meat products and eggs belonged mainly to group A and D [46].

### 3.4. Molecular Typing

**Figure 1:**
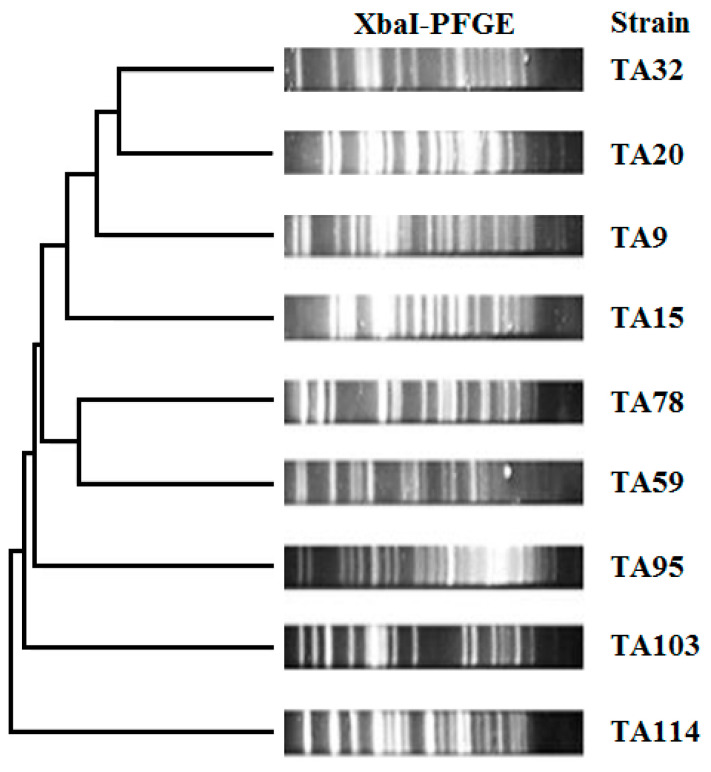
XbaI-PFGE dendrograms showing the genetic relationships of the 9 *mcr-1*-positive *E. coli* strains isolated in this study.

Based on XbaI-PFGE analysis, we found that the nine *mcr-1*-bearing *E. coli* isolates were highly diverse (Figure 1). These data suggested that the spread of *mcr-1* gene among *E. coli* isolates was not due to clonally expansion. MLST analysis result showed that the nine *mcr-1*-bearing *E. coli* isolates belonged to nine STs: ST457, ST69, ST2973, ST469, ST10, ST354, ST3170, ST345, and ST410 (Table 2), which also revealed the high genetic diversity among the nine *mcr-1*-bearing *E. coli* isolates. As the most common *mcr-1*-containing *E. coli*, ST10 was often found in China [47,48]. The *E. coli* ST410 was widely disseminated in the environment, food animals, humans, and wildlife [49]. The high genetic diversity of the *mcr-1*-bearing *E. coli* isolates in this study indicates that the molecular type of *E. coli* isolates from healthy meat ducks is very complicated.

### 3.5. Genetic Environment of Mcr-1 Gene

**Figure 2:**
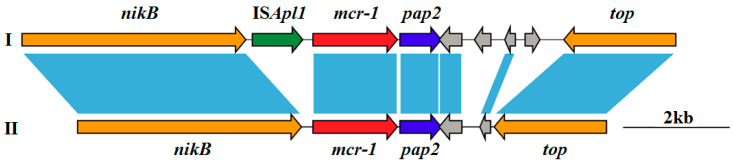
Schematic representation of sequences flanking *mcr-1* gene. Genes and their corresponding transcriptional orientations are indicated by horizontal broad arrows. (**I**) One copy of IS*Apl1* was present beside *mcr-1*; (**II**) no IS*Apl1* was present beside *mcr-1*.

Two different genetic contexts of *mcr-1* (0 or 1 copy of IS*Apl1* was present beside *mcr-1*) were identified among the nine *mcr-1* positive *E. coli* strains (Figure 2 and Table 3). The type I genetic context of *mcr-1* (one copy of IS*Apl1* was present beside *mcr-1*) was identified in seven *mcr-1*-containing *E. coli* isolates. The type II genetic context of *mcr-1* (IS*ApI1* was absent) was found in two *mcr-1*-bearing *E. coli* strains. All *mcr-1* positive *E. coli* strains included the conserved *mcr-1-pap2* segment, which might be horizontally transferred into various plasmids [50]. An IS*Apl1* element was located upstream of the *mcr-1* gene on seven *mcr-1*-positive isolates. The absence of IS*Apl1* in *mcr-1*-bearing plasmids could be explained by the mobilization of an IS*Apl1* composite transposon to conjugative plasmids, which subsequently lost IS*Apl1* copies [51].

### 3.6. Plasmids Analysis

**Figure 3:**
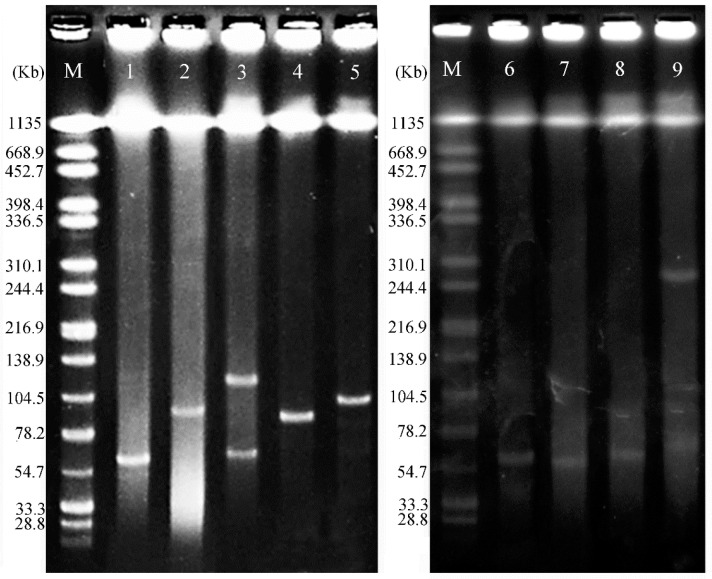
Identification of *mcr-1* gene-carrying plasmids of transconjugants by S1-PFGE. Lanes 1-9: TA15, TA59, TA103, TA78, TA95, TA9, TA20, TA32, TA114. Lane M, *Salmonella* serovar Braenderup H9812.

Conjugation experiments and ERIC-PCR analysis results showed that the colistin-resistant phenotype was successfully transferred from donors to azide-resistant *E. coli* J53 at conjugation frequencies 1.13 × 10^−2^–4.35 × 10^−7^ (transconjugants/recipients) (Table 3). The *mcr-1* gene was identified in 9 transconjugants. S1-PFGE analysis showed that seven transconjugants carried a single plasmid used for plasmid analysis (Figure 3). Transconjugant harbored a single *mcr-1*-associated plasmid, which ranged in size between 65 and 102 kb and was assigned to IncI2 (n = 4), IncFII (n = 2) and IncN (n = 1) replicon types (Table 3), which have been reported by recent studies to be associated with *mcr-1* [14,52,53]. Resistant gene *bla*_CTX-M-55_ was co-transferred with *mcr-1* on pTA59 plasmid, while no other resistant gene was found to coexist with *mcr-1* on the other six plasmids. In this study, two IncI2 plasmids were obtained from the same farm, whereas the other five plasmids were respectively recovered from different farms. As a common *mcr*-disseminator, IncI2 plasmid was identified in isolates from animals, vegetables, and humans [49,54,55]. These results suggest that diversified conjugative plasmids, especially IncI2 plasmid, may be the key vectors that mediate the dissemination of the *mcr-1* among *Enterobacteriaceae* [56]. 

**Figure 4:**
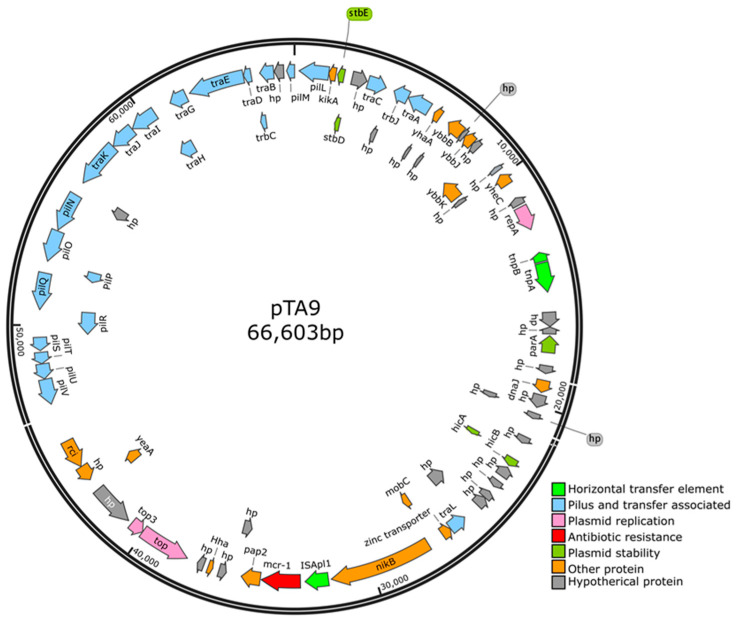
Genomic map of the representative *mcr-1*-carrying plasmid pTA9 from the meat duck gut microbiota.

**Figure 5:**
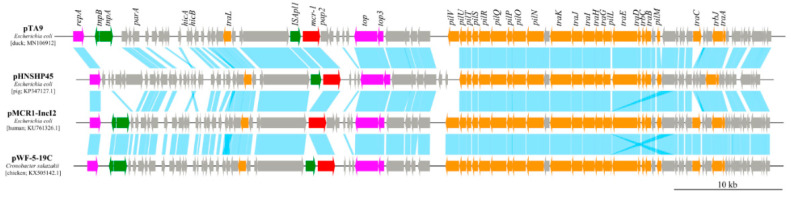
Colinear genome alignments of pTA9 from *E. coli* TA9 isolated in this study, pHNSHP45 from *E. coli* SHP45, pMCR1-IncI2 from *E. coli* SZ02, and pWF-5-19C from *Cronobacter sakazakii* WF-5-19C.

The nucleotide sequence of plasmid pTA9 from strain TA9 has been deposited in GenBank with accession number MN106912. The plasmid size of pTA9 was 66.603 kb, whose GC% was 41.3%, encoding 72 ORFs (Figure 4). The plasmid pTA9 featured an IncI2 plasmid backbone encoding plasmid transfer, stability, and replication. Two conjugative genes (*pil* and *tra*) were predicted on pTA9, which were responsible for the transfer of plasmid between intra- and interspecies bacteria. BLASTn analysis showed that pTA9 was highly similar (the query coverage of 85–97% and the identities 99%) with other *mcr-1*-bearing plasmids, such as pMCR1-IncI2 of *E. coli* (isolated from human in Jiangsu province of China, KU761326.1) [50], pWF-5-19C of *Cronobacter sakazakii* (isolated from chicken in Shandong province of China, KX505142.1) [57], and the first identified *mcr-1*-bearing plasmid pHNSHP45 of *E. coli* (isolated from pig in Shanghai of China, KX505142.1) [14] (Figure 5). *TnpA* and *tnpB* were identified in pTA9, pMCR1-IncI2, and pWF-5-19C. In addition, IS*Apl1* was identified in pTA9, pWF-5-19C, and pHNSHP45. An *mcr-1-pap2* element was identified in pTA9 and pMCR1-IncI2. This suggests that pTA9-like plasmids may be epidemic plasmids that mediate *mcr-1* dissemination between distinct host bacteria in China.

In this study, pTA9 could be transferred to *E. coli* J53 isolates in vitro. This suggests that the *mcr-1* gene present in gut flora of meat duck can be horizontally transferred by bacterial conjugation among distinct bacterial hosts. Similar scenarios have already been observed in the human intestinal flora [58,59]. So *mcr-1*-bearing fecal *E. coli* in healthy meat ducks could be a source for the transfer of *mcr-1* through contaminated food to humans.

## 4. Conclusions

This study revealed the carriage rate of *mcr-1* among fecal *E. coli* isolates obtained from healthy meat ducks in China. PFGE and MLST results indicated that *mcr-1*-bearing *E. coli* isolates were clonally unrelated. This suggested that the horizontal transfer of plasmids was the main mechanism for the dissemination of *mcr-1* gene in meat duck farms. The pTA9-like plasmids have been isolated from different bacterial hosts across distinct regions of China, implying that pTA9-like plasmids are likely to be the epidemic *mcr-1*-bearing plasmids that mediate the dissemination of *mcr-1* in China. Since China is the biggest exporter of meat duck products in the world, the spread of pTA9-like conjugative plasmids across other regions and countries should attract attention. In addition, the *mcr-1*-bearing *E. coli* usually carry *bla*_CTX-M_ and *floR*, conferring resistance to cephalosporins and florfenicol, which made coselection possible when these drugs were used. Restrictive/rational use of antibiotics in animal husbandry, especially in food animals in China may help to limit the spread of *mcr-1* gene.

## Figures and Tables

**Table 1:** The primers used in this study.

Detected Genes		Primer Sequence (5′–3′)	Size/Bp
16S rDNA		agagtttgatcctggctcag	1505
		ggttaccttgttacgactt	
Resistance gene	*rmtB*	atgaacatcaacgatgccctc	756
		ttatccattcttttttatcaagtatat	
Genetic context of the *mcr-1* gene	*nikB*	gatgaacttgatcatcgtgttgt	705
		gtaattctgacgaaaaagacga	
	*top*	gagttcgcaccgctgacagac	330
		atcaaacaccgacttcagggcatc	

**Table 2:** Molecular characteristics of the 9 *mcr-1*-positive *E. coli* strains isolated from healthy meat ducks in this study.

Strains	Farm	MLST	Groups	Virulence Genes	Resistance Genes	Resistant Pattern
TA9 *	1	ST457	A	*iutA*, *papC*	*floR*, *fosA3*	CL/CIP/TET/FFC/FOS ^1^
TA15	2	ST69	A	*iutA*	*bla*_TEM-1_, *fosA3*	CL/CTX/CIP/TET/FOS/AK
TA20	2	ST2973	A	*iutA*	*bla*_CTX-M-55_, *bla*_TEM-1_, *floR*, *fosA3*	CL/CTX/CIP/TET/FFC/FOS
TA32	3	ST469	B1	*iutA*	*bla*_CTX-M-55_, *rmtB*	CL/CTX/CIP/TET/AK
TA59	6	ST10	A	*papC*	*bla*_CTX-M-55_, *floR*, *tet*(A)	CL/CTX/TET/FFC/AK/GN
TA78	8	ST354	A	*papA*	*bla*_TEM-1_, *floR, tet*(A)	CL/CTX/CIP/TET/FFC/GN
TA95	10	ST3170	A	*kpsMT* II	*bla*_TEM-1_	CL/CTX/CIP/TET/FFC
TA103 *	11	ST345	D	*iutA*, *papC*	*bla*_CTX-M-55_, *floR*	CL/CTX/CIP/TET/FFC
TA114	12	ST410	A	*iutA*	*bla*_CTX-M-55_, *bla*_TEM-1_, *floR*, *rmtB*	CL/CTX/CIP/TET/FFC/AK

* The ExPEC strains. ^1^ CL, colistin; FOS, fosfomycin; TET, tetracycline; FFC, florfenicol; CTX, cefotaxime; GN, gentamicin; CIP, ciprofloxacin; AK, amikacin.

**Table 3:** Characterization of some plasmids carrying *mcr-1* of transconjugants.

Strains	Co-Transfer of Other Resistance Gene	Co-Transfer of Virulence Gene	Resistant Patterns	Contest of *Mcr-1*	Conjugation Efficiency	*Mcr-1*-Carrying Plasmids	
						Size (kb)	Replicon Type
TA9 *	/	/	CL ^1^	I	1.13 × 10^−2^	≈65	I2
TA15	/	/	CL	I	6.64 × 10^−4^	≈65	I2
TA20	/	/	CL	II	7.56 × 10^−2^	≈65	I2
TA32	/	/	CL	I	2.17 × 10^−3^	≈65	I2
TA59	*bla*_CTX-M-55_	/	CL/CTX	I	2.98 × 10^−6^	≈102	FII
TA78	/	/	CL	I	1.85 × 10^−5^	≈95	N
TA95	/	/	CL	I	9.93 × 10^−5^	≈102	FII
TA103 *	*bla*_CTX-M-55_, *floR*	/	CL/CTX/FFC	II	4.35 × 10^−7^	/	/
TA114	*floR*	/	CL/FFC	I	3.19 × 10^−6^	/	/

* The ExPEC strains. ^1^ CL, colistin; CTX, cefotaxime; FFC, florfenicol.

## Supplementary Materials

The following are available online at https://www.mdpi.com/2076-2615/10/8/1299/s1, Table S1: The primers used in this study.
